# Isolation and Characterization of Aphidicolin Derivatives from *Tolypocladium inflatum*

**DOI:** 10.3390/molecules22071168

**Published:** 2017-07-12

**Authors:** Jie Lin, Shubin Niu, Zhengfeng Ding, Renlei Wang, Qun Dai, Wei Wei, Rongrong Luo, Ling Liu

**Affiliations:** 1 Jiangsu Key Laboratory for Biofunctional Molecules, College of Life Science and Chemistry,Jiangsu Second Normal University, Nanjing 210003, China; inasianskyjie@aliyun.com (J.L.); ding@jssnu.edu.cn (Z.D.); wrl3501988@163.com (R.W.); njdaiqun@163.com (Q.D.); iamwei.w@163.com (W.W.); 18761602885@163.com (R.L.); 2 School of Biomedicine, Beijing City University, Beijing 100083, China; niushubin@bcu.edu.cn; 3 State Key Laboratory of Mycology, Institute of Microbiology, Chinese Academy of Sciences, Beijing 100101, China

**Keywords:** *Tolypocladium inflatum*, structure elucidation, biological activities

## Abstract

Inflatin G (**1**), a new aphidicolin analogue, together with seven known compounds inflatin A (**2**), inflatin B (**3**), aphidicolin (**4**), aphidicolin-17-monoacetate (**5**), gulypyrone A (**6**), pyridoxatin rotamers A (**7**) and B (**8**), were isolated from the ascomycete fungus *Tolypocladium inflatum*. Their structures were determined through NMR analyses and the circular dichroism data of the in situ formed [Rh_2_(OCOCF_3_)_4_] complexes. Compounds **1**, **4**, **5**, **7**, and **8** showed modest cytotoxicity against four human cancer cell lines A549, CNE1-MP1, A375, and MCF-7.

## 1. Introduction

**Figure 1:**
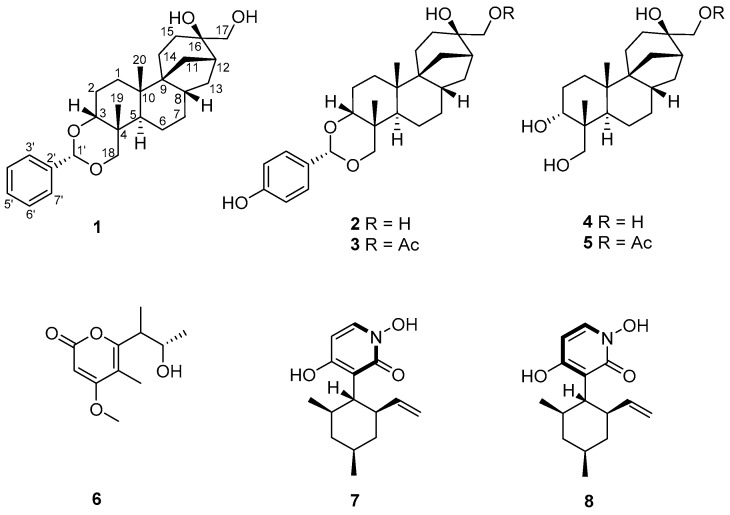
Structures of compounds **1**–**8**.

Fungi which are rich sources of bioactive natural products have played an important role in discovery of lead compounds for therapeutics [1]. Since the secondary metabolism of fungi may be influenced by selection pressures exerted by other organisms and the environment in which they reside, those species thriving in unique and competitive niches are especially likely to produce bioactive natural products with diverse and interesting structural features [2,3]. On the basis of this consideration and the documented success in finding new bioactive natural products from special types of fungi [4], we initiated chemical studies of the *Cordyceps*-colonizing fungi [5] and those that were isolated from the soil samples surrounding *Cordyceps sinensis* [6]. In our study, the ascomycetous fungus *T. inflatum* (SCK6-CP14) was isolated from a soil sample on the surface of *C. sinensis* collected in Kangding, Sichuan, People’s Republic of China. Our prior investigations of *T. inflatum* grown in solid-substrate fermentation cultures have resulted in the isolation of structurally diverse and biologically active metabolites, such as the aphidicolin and chlamydosporol derivatives [6]. Subsequent chemical investigations of the extract from a large-scale fermentation of the strain led to the isolation of a new aphidicolin analogue, which we named inflatin G (Figure 1), along with seven known compounds—inflatin A (**2**) [6], inflatin B (**3**) [6], aphidicolin (**4**) [7], aphidicolin-17-monoacetate (**5**) [8], gulypyrone A (**6**) [9], pyridoxatin rotamers A (**7**) and B (**8**) [10]. All isolate compounds were tested for cytotoxic activities. Herein, the details of the isolation, structural elucidation, and cytotoxicity of these compounds are described.

## 2. Results and Discussion

### 2.1. Isolation and Structure Elucidation of Compound ***1***

**Figure 2:**
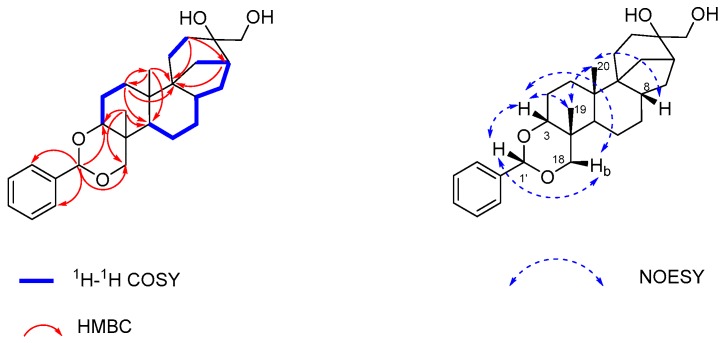
Key ^1^H-^1^H COSY, HMBC and NOESY correlations for **1**.

Inflatin G (**1**) was isolated as a pale yellow oil, and its molecular formula was determined as C_27_H_38_O_4_ (nine degrees of unsaturation) on the basis of the HR-ESI-MS (*m*/*z* 427.2839, calcd. 427.2843 [M + H]^+^). Its NMR data (Table 1) revealed the presence of two exchangeable protons at *δ*_H_ 3.07 and 3.50, respectively, two methyl groups, 10 methylenes (2 oxygenated), 5 methines (2 oxygenated including 1 with double oxygenation at *δ*_C_ 102.4), 6 aromatic/olefinic carbons (5 of which are protonated), and 4 sp^3^ quaternary carbons (1 oxygenated). These data accounted for all the NMR resonances for **1**. Interpretation of its ^1^H-^1^H COSY NMR data (Figure 2 and Figure S3) identified four proton spin-systems corresponding to the C-1–C-3, C-5–C-13 (excluding C-9 and C-10), C-14–C-15 and C-3′-C-7′ fragments. NMR resonances for the five aromatic protons at *δ*_H_ 7.35–7.51 were observed suggesting the presence of a mono-substituted phenyl ring in **1**. HMBC correlations (Figure 2 and Figure S5) from H_2_-1 to C-3 and C-5, H_2_-2 to C-4 and C-10 and H_3_-19 to C-3 and C-5, together with those from H-5 to C-7 and C-9, H-8 to C-6 and C-10, H_3_-20 to C-1, C-5 and C-9 and H_2_-1 to C-9 completed assignment of the decahydronaphthalene moiety. Correlations from H_2_-11 to C-8 and C-10, H-12 to C-8 and C-9 and H_2_-14 to C-8, C-10 and C-11 located the C-9 between C-11 and C-14. Correlations from H_2_-11 and H_2_-13 to C-16, H_2_-17 to C-12 and C-15, H_2_-15 to C-9 and C-12 made C-16 connected to C-12, C-15, and C-17, completing the tetradecahydro-8,11a-methanocyclohepta[*a*]naphthalene skeleton. This substructure of **1** is similar to aphidicolin (**4**) [7], a known compound that was co-isolated as the major component from the crude extract, implying that **1** could be a condensation product of benzaldehyde and **4**. These observations were supported by HMBC correlations from the acetal proton (H-1′; *δ*_H_ 5.51) to C-3, C-18, C-3′, and C-7′. The two exchangeable protons in **1** were assigned by default as the C-16 and C-17 hydroxy groups, respectively. On the basis of these data, the planar structure of **1** was established as shown.

**Figure 3:**
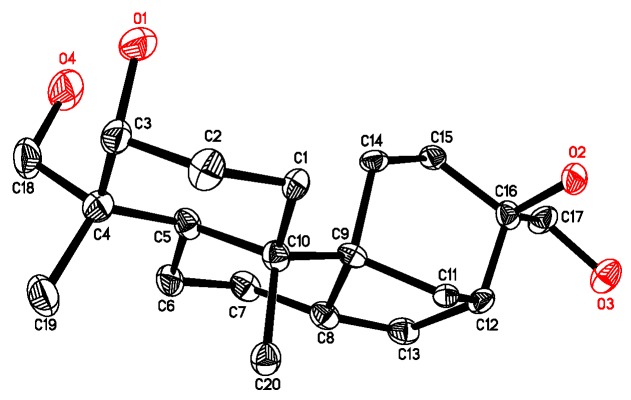
Thermal ellipsoid representation of **4**.

The relative configuration of **1** was determined on the basis of NOESY data (Figure S6) and by comparison with that of aphidicolin (**4**). NOESY correlations of H-1′ with H-3 and H-18b, H-3 with H-18b and H_3_-19, H_3_-19 with H_3_-20, H_3_-20 with H-8 revealed their proximity in space (Figure 2). The remaining portion of **1** was deduced to have the same relative configuration as their counterparts in **4** [7], which was confirmed by single-crystal X-ray diffraction analysis (Figure 3).

**Figure 4:**
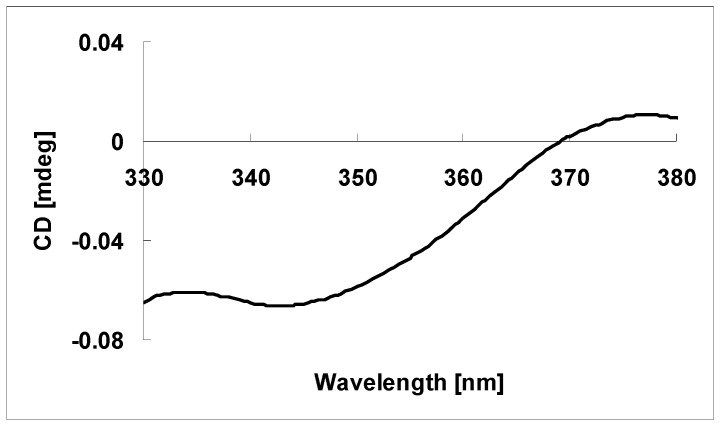
CD spectrum of Rh-complex of **1** with the inherent CD spectrum subtracted.

The absolute configuration at C-16 in **1** was determined on the basis of the circular dichroism data of the complex formed in situ Rh_2_(OCOCF_3_)_4_ [11,12], with the inherent contribution subtracted. Upon addition of Rh_2_(OCOCF_3_)_4_ to a solution of **1** in CH_2_Cl_2_, a metal complex was generated, acting as an auxiliary chromophore. It has been demonstrated that the sign of the E band (at ca. 350 nm) can be used to correlate the absolute configuration of a tertiary alcohol by applying the bulkiness rule [11,12]. In this case, the Rh-complex of **1** displayed a negative E band (Figure 4), correlating to the 16*R* absolute configuration. This assignment is consistent with that reported for inflatin A (**2**) [6]. Therefore, the 3*R*, 4*R*, 5*R*, 8*S*, 9*S*, 10*S*, 12*R*, 16*R*, 1′*S* absolute configuration was assigned for **1**.

By comparison of the NMR and MS data with those reported, compounds **2**–**8** isolated from the crude extract were identified as inflatin A (**2**), inflatin B (**3**), aphidicolin (**4**), aphidicolin-17-monoacetate (**5**), gulypyrone A (**6**), pyridoxatin rotamers A (**7**) and B (**8**), respectively.

### 2.2. Bioactivities

Compounds **1**, **4**–**8** were tested for the cytotoxicities against A549, CNE1-MP1, A375, and MCF-7 human cancer cell lines using MTS method. Compound **1** showed the modest cytotoxic effects against MCF-7 cells, with IC_50_ values of 35.2 µM, while the positive control paclitaxel showed an IC_50_ value of 1.4 × 10^−2^ µM (Table 2). Compound **7** and **8** displayed cytotoxicity against A375 cells, with an IC_50_ value of 0.10 µM. Compounds **1**, **4**–**8** also showed cytotoxicity against the negative control HaCaT (human keratinocyte) cells, implying the lack of selectivity for these metabolites.

## 3. Experimental Section

### 3.1. General Experimental Procedures

Optical rotations were measured on a 241 polarimeter (Perkin-Elmer, Norwalk, CT, USA), and UV data were recorded on a Shimadzu Biospec-1601 spectrophotometer (Shimadzu, Kyoto, Japan). CD spectra were recorded on a JASCO J-815 spectropolarimeter (JASCO, Tokyo, Japan). IR data were recorded using a Nicolet Magna-IR 750 spectrophotometer (Nicolet, Madison, WI, USA). ^1^H- and ^13^C-NMR data were acquired with Varian Mercury-500 spectrometers (Varian, Palo Alto, CA, USA) using solvent signals (acetone-*d*_6_: *δ*_H_ 2.05/*δ*_C_ 29.8, 206.1) as references. The HSQC and HMBC experiments were optimized for 145.0 and 8.0 Hz, respectively. ESIMS data were recorded on a Bruker Esquire 3000^plus^ (Bruker, Billerica, MA, USA) spectrometer, and HRESIMS data were obtained using Bruker APEX III 7.0T (Bruker, Billerica, MA, USA) and APEXII FT-ICR spectrometers (Bruker, Billerica, MA, USA), respectively.

### 3.2. Fungal Material

*T. inflatum* was collected in Kangding, the southwest of China, in May 2005. Although *T. inflatum* is chiefly as a soil fungus, its sexual state has been encountered as a pathogen of insects, specifically beetle larvae. According to morphology and sequence (Genbank Accession No. JN003828) analysis of the ITS region of the rDNA, the isolate was identified and assigned the accession number SCK6-CP14 in culture collection at the Institute of Microbiology, Chinese Academy of Sciences, Beijing. The fungal strain was cultured on slants of potato dextrose agar (PDA) at 25 °C for 10 days. Agar plugs were cut into small pieces (about 0.5 × 0.5 × 0.5 cm^3^) under aseptic conditions, and 15 pieces were used to inoculate three Erlenmeyer flasks (250 mL), each containing 50 mL of media (0.4% glucose, 1% malt extract, and 0.4% yeast extract); the final pH of the media was adjusted to 6.5 and sterilized by autoclave. Three flasks of the inoculated media were incubated at 25 °C on a rotary shaker at 170 rpm for five days to prepare the seed culture. Fermentation was carried out in eight Fernbach flasks (500 mL), each containing 80 g of rice. Spore inoculum was prepared by suspension in sterile, distilled H_2_O to give a final spore/cell suspension of 1 × 10^6^/mL. Distilled H_2_O (120 mL) was added to each flask, and the contents were soaked overnight before autoclaving at 15 psi for 30 min. After cooling to room temperature, each flask was inoculated with 5.0 mL of the spore inoculum and incubated at 25 °C for 40 days.

### 3.3. Extraction and Isolation

The fermented material (eight Fernbach flasks) was pounded with glass rod and extracted with EtOAc (4 ×1.0 L). The solid phase was separated from EtOAc using separating funnel with filter paper. The resulting solution was evaporated to dryness under vacuum at 35 °C on a rotavapor (eyela) to afford the crude extract (8.7 g), which was fractionated by silica gel VLC using petroleum ether–EtOAc gradient elution.

The fraction (154 mg) eluted with 30% EtOAc was separated by Sephadex LH-20 column chromatography (CC) eluting with 1:1 CH_2_Cl_2_–MeOH. The resulting subfractions were combined and further purified by semipreparative RP HPLC (Agilent Zorbax SB-C_18_ column; 5 μm; 9.4 × 250 mm; 30% MeOH in H_2_O for 5 min, followed by 30–70% over 40 min; 2 mL/min) to afford mixtures of **7** and **8** (7.5 mg, *t*_R_ 17.20 min).

The fraction (146 mg) eluted with 35% EtOAc was separated by Sephadex LH-20 CC eluting with 1:1 CH_2_Cl_2_–MeOH. The resulting subfractions were combined and purified by RP HPLC (Agilent Zorbax SB-C_18_ column; 5 μm; 9.4 × 250 mm; 70% MeOH in H_2_O for 5 min, followed by 70–95% over 35 min; 2 mL/min) to afford **1** (7.3 mg, *t*_R_ 26.40 min), **2** (20.0 mg, *t*_R_ 28.32 min), and **3** (7.2 mg, *t*_R_ 32.31 min).

The fraction (110 mg) eluted with 50% EtOAc was separated by Sephadex LH-20 CC eluting with MeOH, and the resulting subfractions were purified by RP HPLC (68% MeOH in H_2_O for 2 min, followed by 68–84% over 40 min; 2 mL/min) to afford **4** (1.8 mg, *t*_R_ 36.21 min) and **5** (1.8 mg, *t*_R_ 36.21 min).

The fraction (155 mg) eluted with 45% EtOAc was separated by Sephadex LH-20 CC eluting with MeOH, and the resulting subfractions were purified by RP HPLC (20% MeOH in H_2_O for 2 min, followed by 20–62% over 38 min; 2 mL/min) to afford **6** (1.8 mg, *t*_R_ 36.21 min).

### 3.4. Spectroscopic Data

Inflatin G (**1**): pale yellow oil; [α]23D +3.3 (*c* 2.1, MeOH); UV (MeOH) λ_max_ (log ε) 205 (3.53), 210 (3.76), 260 (2.67), 287 (2.40) nm; CD (*c* 6.0 × 10^−4^ M, CH_2_Cl_2_) λ_max_ (Δε) 218 (–0.04), 237 (+0.07); IR (neat) ν_max_ 3396 (br), 2935, 2861, 1455, 1393, 1157, 1121, 1102 cm^−1^; ^1^H-, ^13^C-NMR, and HMBC data see Table 1; NOESY correlations (acetone-*d*_6_, 500 MHz) H-3 ↔ H-18b, H_3_-19, H-1′; H-5 ↔ H-18a; H-8 ↔ H_3_-20; H-18a ↔ H-5; H-18b ↔ H-3, H_3_-19, H-1′; H_3_-19 ↔ H-3, H-18b, H_3_-20; H_3_-20 ↔ H-8, H_3_-19; H-1′ ↔ H-3, H-18b; HRESIMS *m/z* 427.2839 (calcd. for C_27_H_39_O_5_, 427.2843).

Inflatin A (**2**): ^1^H-, ^13^C-NMR, and the MS data were fully consistent with literature values [6].

Inflatin B (**3**): ^1^H-, ^13^C-NMR, and the MS data were fully consistent with literature values [6].

Aphidicolin (**4**): ^1^H-, ^13^C-NMR, and the MS data were fully consistent with literature values [7].

X-ray crystallographic analysis of aphidicolin (**4**) [13]. Upon crystallization from acetone–H_2_O (4:1) using the vapor diffusion method, colorless crystals were obtained for **4**, a crystal (0.24 × 0.23 × 0.15 mm ) was separated from the sample and mounted on a glass fiber, and data were collected using a Rigaku RAPID IP diffractometer with graphite-monochromated Mo Kα radiation, λ = 0.71073 Ǻ at 173(2) K. Crystal data: C_20_H_34_O_4_, M = 338.47, space group tetragonal, P4(3)2(1)2; unit cell dimensions *a* = 11.7752 (17) Ǻ, *b* = 11.7752 (17) Ǻ, *c* = 25.579 (5) Ǻ, V = 3546.6(10) Ǻ^3^, Z = 8, D_calcd._ = 1.268 mg/m^3^, µ = 0.086 mm^−1^, F(000) = 1488. The structure was solved by direct methods using SHELXL-97 [14] and refined by using full-matrix least-squares difference Fourier techniques. All non-hydrogen atoms were refined with anisotropic displacement parameters and all hydrogen atoms were placed in idealized positions and refined as riding atoms with the relative isotropic parameters. Absorption corrections were performed using the Siemens Area Detector Absorption Program (SADABS) [15]. The 20669 measurements yielded 2143 independent reflections after equivalent data were averaged, and Lorentz and polarization corrections were applied. The final refinement gave R_1_ = 0.0956 and wR_2_ = 0.1702 (I > 2σ(I)).

Aphidicolin-17-monoacetate (**5**): ^1^H-, ^13^C-NMR, and the MS data were fully consistent with literature values [8].

Gulypyrone A (**6**): ^1^H-, ^13^C-NMR, and the MS data were fully consistent with literaturevalues [9].

Pyridoxatin rotamers A (**7**) and B (8): ^1^H-, ^13^C-NMR, and the MS data were fully consistent with literature values [10]. 

### 3.5. Absolute Configuration of the Tertiary Alcohol in ***1*** [11,12]

According to the published procedure [11,12], a sample of **1** (0.5 mg) was dissolved in a dry solution of the stock Rh_2_(OCOCF_3_)_4_ complex (1.5 mg) in CH_2_Cl_2_ (200 μL). The first CD spectrum was recorded immediately after mixing, and its time evolution was monitored until stationary (about 10 min after mixing). The inherent CD was subtracted. The observed sign of the E band at ca. 350 nm in the induced CD spectrum was correlated to the absolute configuration of the C-16 tertiary alcohol moiety.

### 3.6. Cytotoxicity Assay [16,17]

Cytotoxic activity was tested against four human cancer cell lines (A549, CNE1-MP1, A375, and MCF-7) using the MTS method. For the MTS assay, the CellTiter 96**^®^** AQueous One Solution Reagent (Promega, Madison, WI, USA) was used following the manufacturer’s instruction. In a 96-well plate, each well was plated with 2–5 × 10^3^ cells (It depends on the cell multiplication rate). After cell attachment overnight, the medium was removed, and each well was treated with 100 μL of medium containing 0.1% DMSO, or appropriate concentrations of the test compounds and the positive control paclitaxel (Sigma, St. Louis, MI, USA) (100 mM as stock solution of a compound in DMSO and serial dilutions; the test compounds showed good solubility in DMSO and did not precipitate when added to the cells). The plate was incubated for 72 h at 37 °C in a humidified, 5% CO_2_ atmosphere. Proliferation assessed by adding 20 μL of MTS to each well in dark followed by a 90 min incubation at 37 °C. The assay plate was read at 490 nm using a microplate reader (Bio-Rad, Hercules, CA, USA). The assay was run in triplicate.

## 4. Conclusions

The present study aimed at identifying active novel compounds from fungi which may be developed into more effective and affordable drugs. From this study, inflatin G (**1**), a new aphidicolin analogue, together with seven known compounds, were isolated from the ascomycete fungus *T. inflatum*. Their structures were determined through NMR analyses and the circular dichroism data of the in situ formed Rh_2_(OCOCF_3_)_4_ complexes. Inflatin G (**1**) which possesses a 1,3-dioxane moiety *cis* fused to the aphidicolin core at C-3/C-4 like that found in the natural precedent aphidicolin-3,18-orthoacetate, is structurally related to the known fungal metabolite inflatin A [6], but differs in having a mono-substituted phenyl ring in **1** rather than a *p*-substituted phenyl ring in inflatin A. These compounds also showed modest cytotoxicity against four human cancer cell lines. However, further investigations should be conducted to explore its action mechanism and safety issues, so as to develop a fundamental structure with potential bioactivities. In this work, the discovery of new aphidicolin derivative further expanded the structural diversity of the secondary metabolites produced by *T. inflatum*.

## Figures and Tables

**Table 1:** NMR Data for **1** in Acetone-*d*_6._

Pos.	*δ*_C_ *^a^*, Mult.	*δ*_H_ *^b^* (*J* in Hz)	HMBC *^b^*
1a	27.9, CH_2_	2.20, td (11.0, 3.0)	9, 20
1b		0.96, dt (11.0, 2.5)	3, 5
2a	27.8, CH_2_	1.67–1.72 *^c^*	4, 10
2b		1.30–1.36 *^c^*	
3	81.8, CH	3.66, t (3.0)	1, 5, 18, 19, 1′
4	35.7, qC		
5	34.6, CH	2.82, dd (10.5, 2.5)	1, 7, 9, 19, 20
6a	23.2, CH_2_	1.63, m	8
6b		1.28–1.36 *^c^*	
7a	25.2, CH_2_	1.92–2.02 *^c^*	9
7b		1.68–1.70 *^c^*	5, 9
8	41.1, CH	1.96–2.08 *^c^*	6, 10, 12
9	49.9, qC		
10	40.6, qC		
11a	33.4, CH_2_	1.89–1.91 *^c^*	8, 10, 13, 14, 16
11b		1.25–1.37 *^c^*	10, 14, 16
12	42.3, CH	2.13, t (5.7)	8, 9, 15, 17
13a	31.9, CH_2_	1.70–1.72 *^c^*	9, 16
13b		0.99, dd (11.5, 7.0)	11, 16
14a	25.5, CH_2_	1.97–2.12 *^c^*	8, 10, 11, 16
14b		1.71–1.76 *^c^*	8, 10, 16
15a	29.3, CH_2_	1.48, dt (12.0, 2.7)	17
15b		1.30, td (12.0, 5.0)	9, 12
16	74.3, qC		
17a	68.3, CH_2_	3.37, dd (9.0, 5.0)	12, 15
17b		3.27, dd (9.0, 5.0)	12, 15
18a	76.2, CH_2_	4.02, d (10.0)	3, 1′
18b		3.16, d (10.0)	3, 5, 19, 1′
19	17.2, CH_3_	0.76, s	3, 5, 18
20	16.0, CH_3_	1.05, s	1, 5, 9
1′	102.4, CH	5.51, s	3, 18, 3′, 7′
2′	140.6, qC		
3′	127.3, CH	7.51, d (6.5)	1′, 5′, 7′
4′	128.8, CH	7.35, d (6.5)	2′, 6′
5′	129.3, CH	7.36, m	
6′	128.8, CH	7.35, d (6.5)	2′, 4′
7′	127.3, CH	7.51, d (6.5)	1′, 3′, 5′
OH-16		3.07, s	
OH-17		3.50, t (5.0)	

*^a^* Recorded at 125 MHz; *^b^* Recorded at 500 MHz; *^c^* Signals overlapping.

**Table 2:** Cytotoxicity of Compounds **1**, **4**–**8** Against Human Tumor Cell Lines.

Compound	IC_50_ (µM)				
	A549 *^a^*	CNE1-LMP1 *^b^*	A375 *^c^*	MCF-7 *^d^*	HaCaT *^e^*
**1**	68.7	35.4	39.8	35.2	42.6
**4**	16.2	3.12	37.3	42.3	10.4
**5**	5.20	4.00	40.5	66.2	8.00
**6**	>100	>100	>100	>100	>100
**7** and **8**	0.44	0.20	0.10	0.17	>47.5
paclitaxel	3.0 × 10^−2^	4.2 × 10^−3^	8.9 × 10^−3^	1.4 × 10^−2^	0.024

*^a^* Lung adenocarcinoma cells; *^b^* Stable oncoprotein LMP1 integrated nasopharyngeal carcinoma cells; *^c^* Malignant melanoma cells; *^d^* Breast cancer cells; *^e^* Keratinocyte cells.

## Supplementary Materials

Supplementary materials are available online: NMR spectra data (Figure S1–S6) of compound **1** as supporting information.
