# Supplementary material for: Isolation and Characterization of Aphidicolin Derivatives from Tolypocladium inflatum
Source: Molecules. 2017 Jul 12;22(7):1168. doi: 10.3390/molecules22071168 (PMC6152040; doi:10.3390/molecules22071168)

*Molecules* **2017**, *x*,1-x manuscripts; doi:10.3390/molecules190x0000x

**OPEN ACCESS**

***molecules***

**ISSN 1420-3049**

[www.mdpi.com/journal/](http://www.mdpi.com/journal/)molecules

*Article*

**Isolation and Characterization of Aphidicolin Derivatives from *Tolypocladium inflatum***

**Jie Lin,^1,∆^ Shubin Niu,^2,∆^ Zhengfeng Ding,^1^ Renlei Wang,^1^ Qun Dai,^1^ Wei Wei,^1^ Rongrong Luo^1^ and Ling Liu^3,*^**

^1^ Jiangsu Key Laboratory for Biofunctional Molecules, College of Life Science and Chemistry, Jiangsu Second Normal University, Nanjing, People’s Republic of China

^2^ School of Biomedicine, Beijing City University, Beijing, People's Republic of China

^3^ State Key Laboratory of Mycology, Institute of Microbiology, Chinese Academy of Sciences, Beijing, People’s Republic of China

***** Author to whom correspondence should be addressed; E-Mails: liul@im.ac.cn; Tel.: +86 10 64806153.

∆ Contributed equally to this work.

Academic Editor: name

Received: date; Accepted: date; Published: date

**Contents Page**

1) **Figure S1.** ^1^H NMR spectrum of inflatin G (**1**; 500 MHz, acetone-*d*_6_) 2

2) **Figure S2.** ^13^C NMR spectrum of inflatin G (**1**; 125 MHz, acetone-*d*_6_) 3

3) **Figure S3.** ^1^H-^1^H COSY spectrum of inflatin G (**1**; 500 MHz, acetone-*d*_6_) 4

4) **Figure S4.** HSQC spectrum of inflatin G (**1**; 500 MHz, acetone-*d*_6_) 5

4) **Figure S5.** HMBC spectrum of inflatin G (**1**; 500 MHz, acetone-*d*_6_) 6

3) **Figure S6.** NOESY spectrum of inflatin G (**1**; 500 MHz, acetone-*d*_6_) 7

**Figure S1.** ^1^H NMR Spectrum of Inflatin G (**1**; 500 MHz, Acetone-*d*_6_)


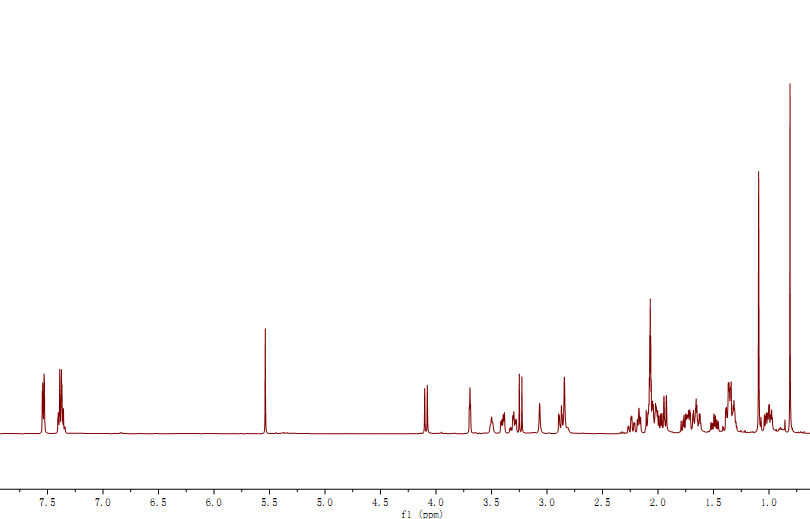


**Figure S2.** ^13^C NMR Spectrum of Inflatin G (**1**; 125 MHz, Acetone-*d*_6_)


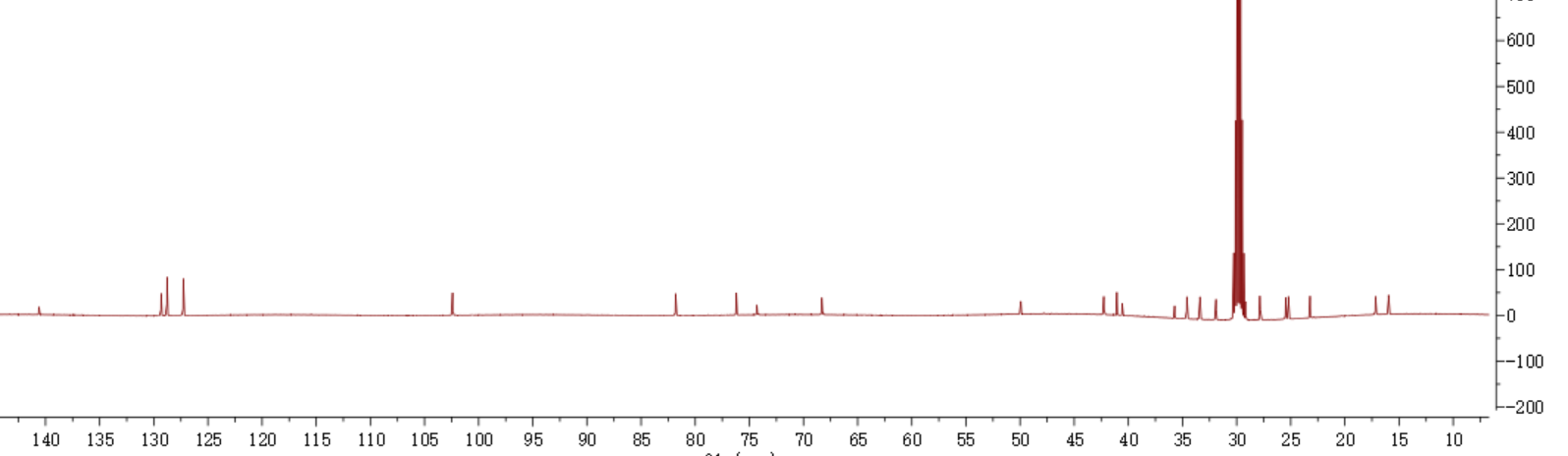


**Figure S3.** ^1^H-^1^H COSY spectrum of inflatin G (**1**; 500 MHz, acetone-*d*_6_)


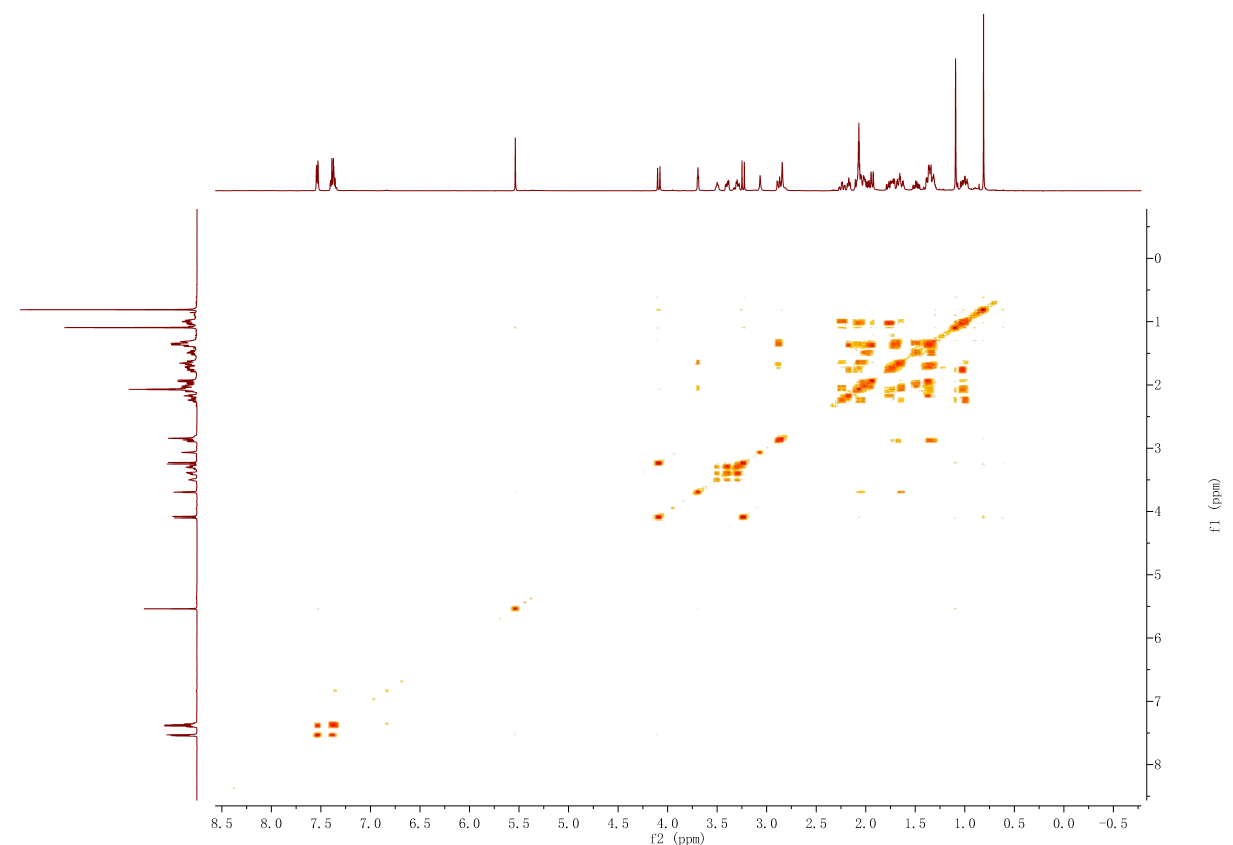


**Figure S4.** HSQC spectrum of inflatin G (**1**; 500 MHz, acetone-*d*_6_)


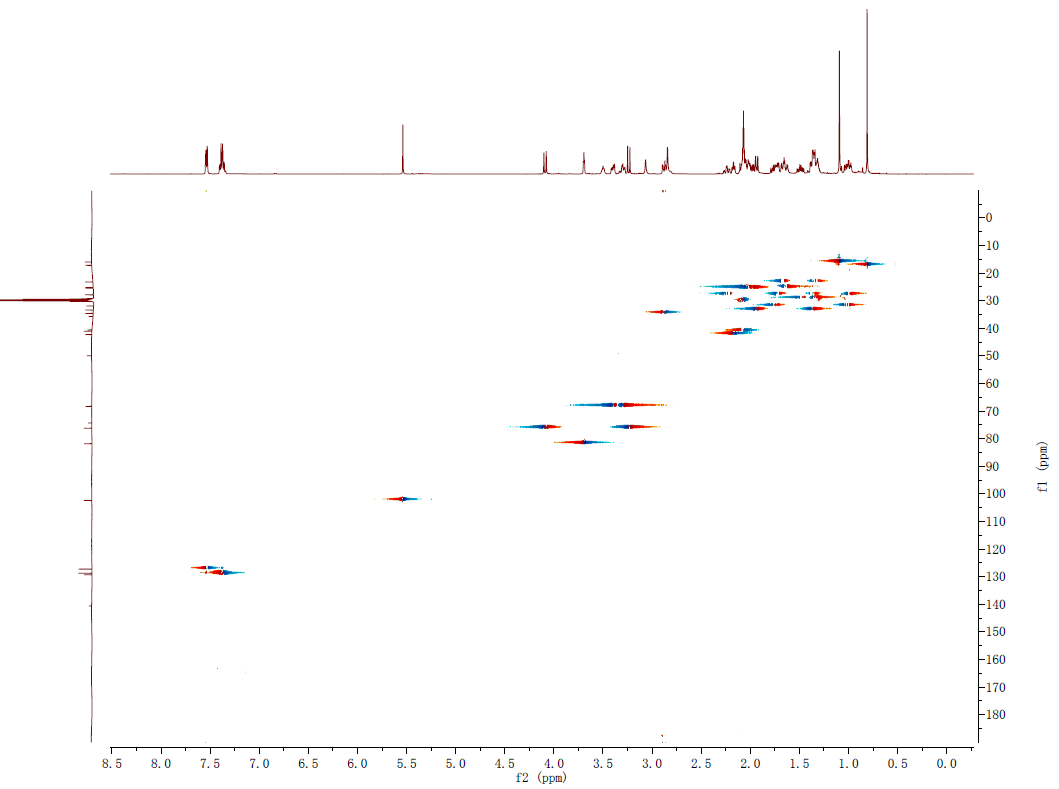


**Figure S5.** HMBC spectrum of inflatin G (**1**; 500 MHz, acetone-*d*_6_)

**
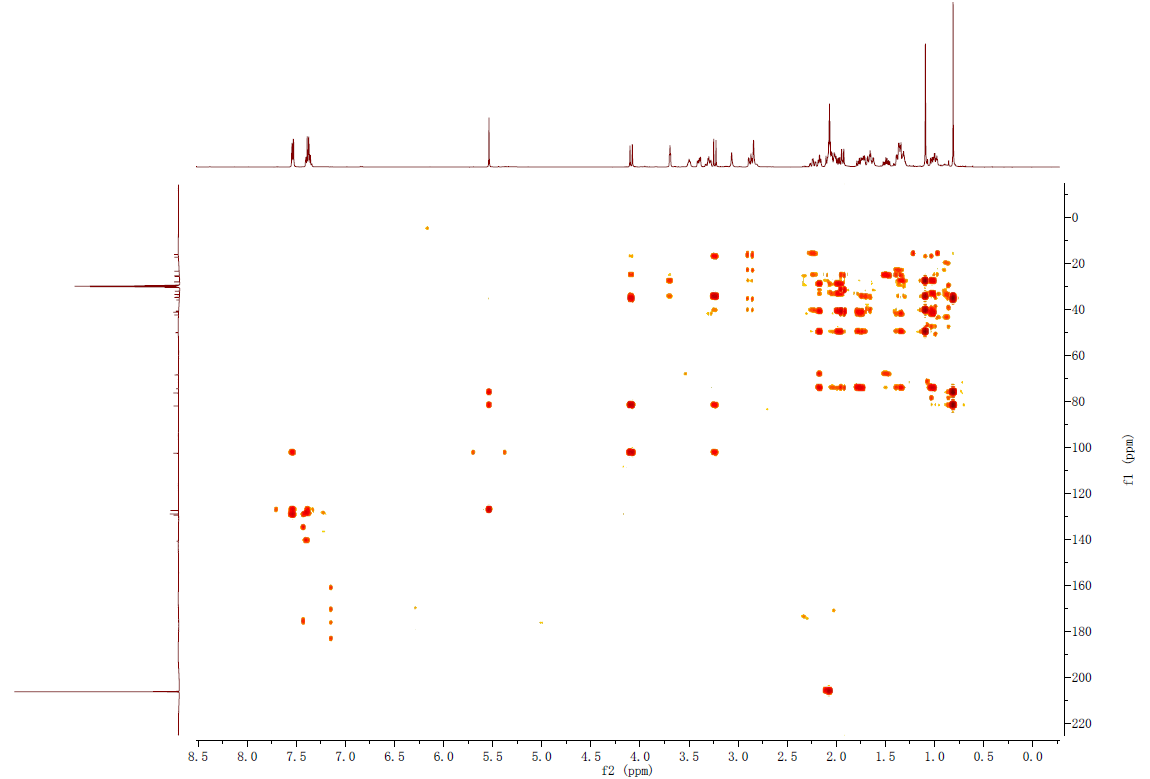
**

**Figure S6.** NOESY spectrum of inflatin G (**1**; 500 MHz, acetone-*d*_6_)


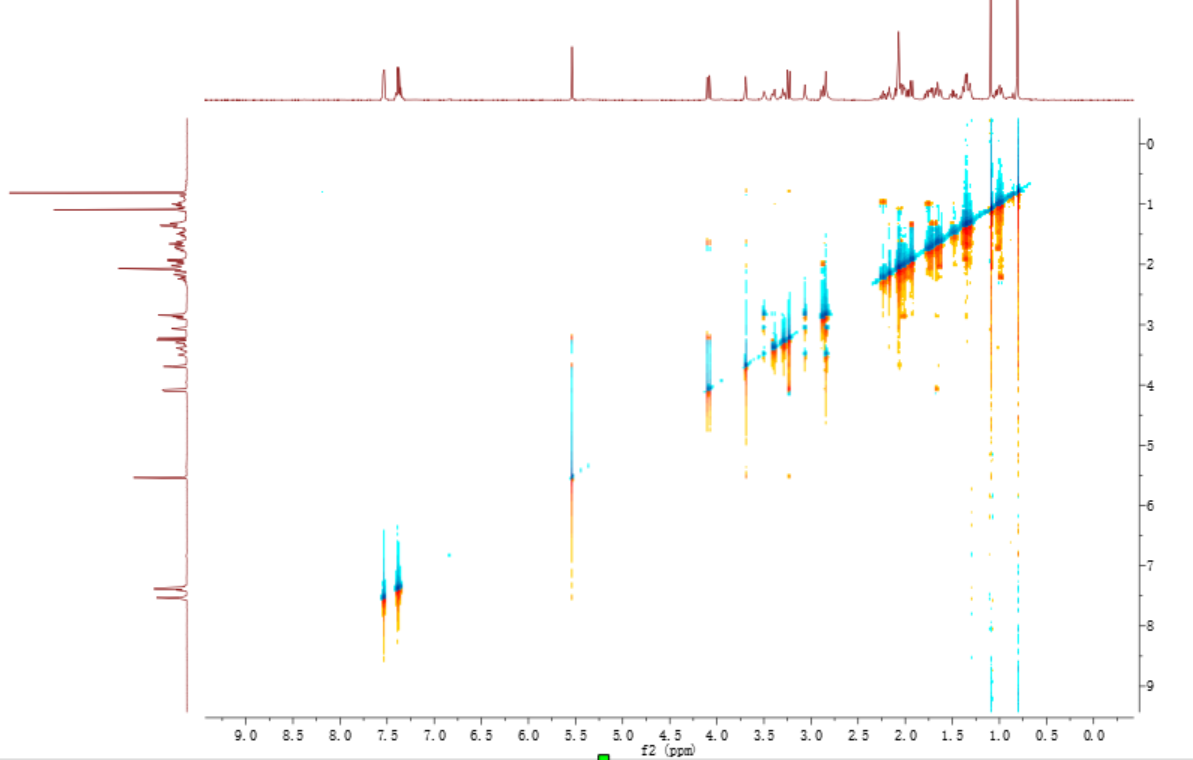

Supplement: Supplementary file 1 [file molecules-22-01168-s001.zip › supporting information/molecules-supporting information.docx]
